# Comparative analysis of four nutritional scores in predicting delirium in ICU patients

**DOI:** 10.3389/fnut.2025.1482150

**Published:** 2025-07-22

**Authors:** Chunchun Yu, Lefu Chen, Xiong Lei, Zhixiao Xu, Hongjun Zhao, Chengshui Chen

**Affiliations:** ^1^Key Laboratory of Interventional Pulmonology of Zhejiang Province, Department of Pulmonary and Critical Care Medicine, The First Affiliated Hospital of Wenzhou Medical University, Wenzhou, China; ^2^Department of Internal Medicine, Nassau University Medical Center, East Meadow, New York, NY, United States; ^3^Department of Pulmonary and Critical Care Medicine, Zhejiang Province Engineering Research Center for Endoscope Instruments and Technology Development, Quzhou People’s Hospital, The Quzhou Affiliated Hospital of Wenzhou Medical University, Quzhou, China

**Keywords:** prognostic nutritional index, geriatric nutritional risk index, triglycerides × total cholesterol × body weight index, controlling nutritional status, delirium

## Abstract

**Background:**

The nutritional assessment indicators for critically ill patients are diverse, with limited research about comparing the predicting value of different nutritional assessment tools for delirium in the intensive care unit (ICU).

**Objectives:**

The study aimed to validate the relationship between malnutrition and ICU delirium and explore the optimal nutritional scores for predicting ICU delirium.

**Methods:**

This study was based on the Medical Information Mart for Intensive Care IV (MIMIC-IV) database and included 319 ICU patients who met the inclusion and exclusion criteria. The study used four nutritional assessment tools: Geriatric Nutritional Risk Index (GNRI), Prognostic Nutritional Index (PNI), Triglycerides (TG) × Total Cholesterol (TC) × Body Weight (BW) Index (TCBI), and Controlling Nutritional Status (CONUT) score. Restricted cubic spline (RCS) modeling, single-factor logistic regression, and multivariate stepwise logistic regression were employed to elucidate the relationships between each nutritional score and delirium. Using area under the curve (AUC) evaluated the discriminatory ability of the adjusted models.

**Results:**

The RCS shows a strong linear connection between delirium and PNI (*P* for nonlinear = 0.66), as well as between delirium and CONUT score (*P* for nonlinear = 0.32). Multivariate logistic regression reveals that PNI (OR = 2.04, 95% CI: 1.05–4.03, *p* = 0.04) has the closest relationship with ICU delirium. The AUC of the PNI prediction model after adjusting covariates was 0.87 (95% confidence interval: 0.83–0.91, *p* < 0.05).

**Conclusion:**

The study confirmed the association between poor nutritional status and increased risk of ICU delirium in patients. PNI demonstrated excellent independent predictive value for ICU delirium, warranting further clinical application and validation.

## 1 Introduction

Delirium primarily manifests as acute brain dysfunction, characterized by rapid changes in consciousness, cognitive function, and attention over a short period (1). The intensive care unit (ICU) serves as a high-risk environment for delirium in patients. The incidence of ICU delirium ranges from 21 to 64%, with a recent meta-analysis reported citing an approximate prevalence rate of around 30% (2). ICU delirium is associated with prolonged hospital stays, long-term cognitive decline, increased risk of mortality, deterioration in quality of life, and significant healthcare costs (3, 4). Despite this, over half of delirium cases are still overlooked in clinical practice (1, 3).

The etiology of delirium is complex and comprises a combination of various predisposing factors. However, 30–40% of delirium cases are preventable, underscoring the importance of early identification and intervention targeting modifiable risk factors to prevent the onset and progression of delirium (5). Increasing evidence supports malnutrition as a potential contributing factor to delirium (6–12). Although most studies support the association between malnutrition and delirium, a study by Zhang et al. (13) did not find a correlation between delirium occurrence and malnutrition. Nutritional issues are commonplace in critically ill inpatients, yet incorporating nutritional considerations as a direct therapeutic strategy for delirium warrants further attention.

NRS-2002 and mNUTRIC are guideline-recommended tools for screening nutritional risk in critically ill patients, though high-quality evidence supporting their application in this population remains limited (14). Additionally, these tools involve complex assessments and may require a level of patient comprehension that can be challenging in ICU settings. In contrast, the Geriatric Nutritional Risk Index (GNRI), Prognostic Nutritional Index (PNI), and Controlling Nutritional Status (CONUT) score are simpler, more objective, and easier-to-use nutritional scoring tools developed in recent years. Previous studies have used GNRI, PNI, and CONUT score to explore the relationship between nutritional status and delirium in critically ill patients, though results have been inconsistent (7, 15). The Triglyceride (TG) × Total Cholesterol (TC) × Body Mass Index (TCBI) is a novel nutritional/intrinsic metabolism index. Unlike NRI, PNI, and CONUT score, TCBI has a simpler calculation; however, its relationship with delirium remains unclear. Although several studies have compared these four nutritional scoring tools (16, 17), research specifically focusing on identifying the optimal nutritional score for predicting ICU delirium is still limited (18).

Therefore, this study selected four simple and objective nutritional scores to assess the correlation between nutritional status in critically ill patients and the occurrence of delirium, including the GNRI, PNI, TCBI, and CONUT score, aiming to provide new insights for the clinical prevention and management of ICU delirium.

## 2 Materials and methods

### 2.1 Data source and participant selection

**Figure 1:**
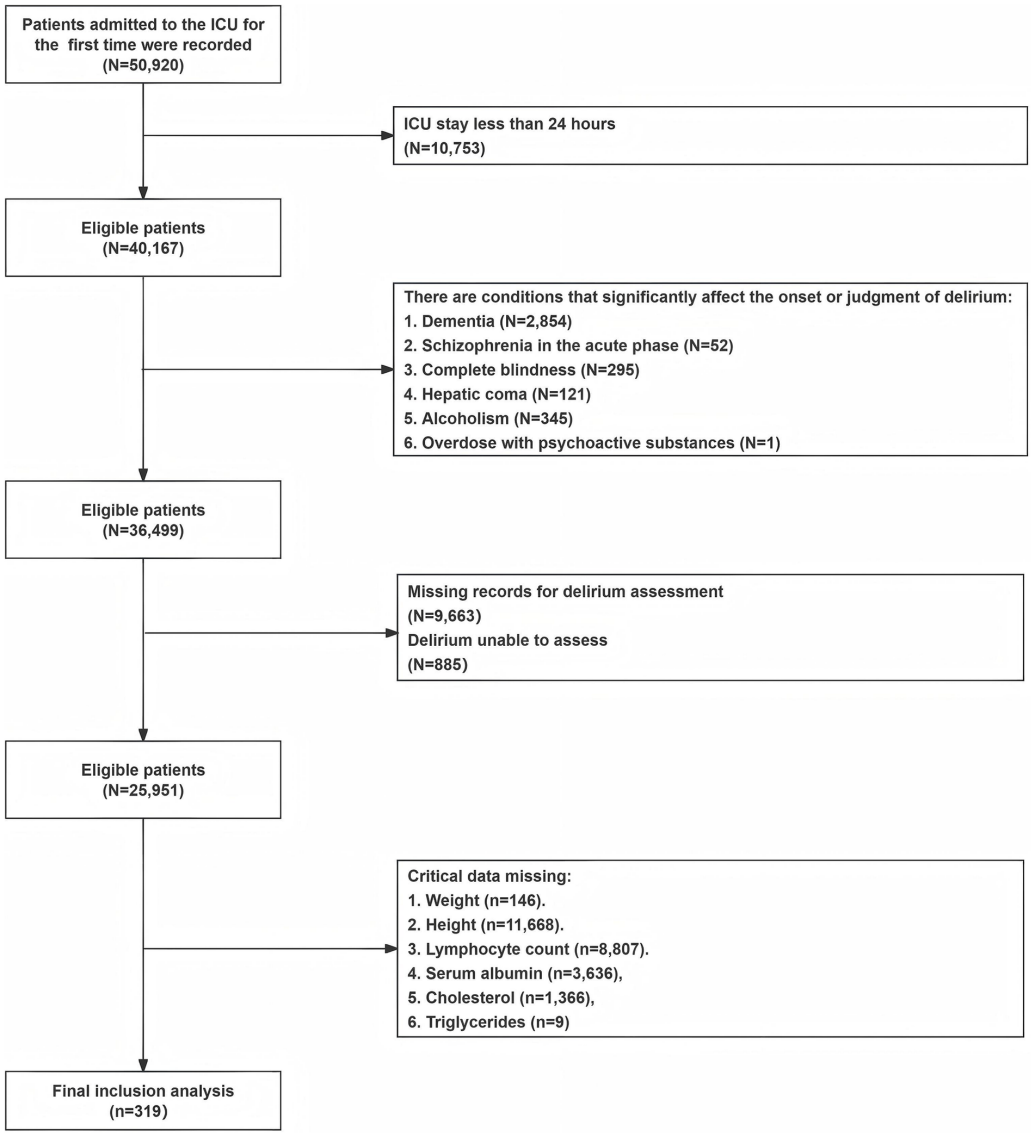
Study flowchart. ICU, intensive care unit.

This study retrospectively analyzed the clinical data of 319 ICU inpatients who met the inclusion and exclusion criteria in the Medical Information Mart for Intensive Care IV database version 2.2 (MIMIC-IV v2.2) (ID for accessing the data: 62506785). The MIMIC-IV database boasts a large population size, high data quality, and open access, providing valuable clinical information for medical research. The study used SQL queries in Navicat Premium (16.3.8 version) to retrieve records and data that met the criteria, with the project barcode for CAM-ICU being 228,332. The records in MIMIC-IV had undergone de-identification, exempting the need for individual patient written informed consent. Inclusion criteria for participants were as follows: 1. First ICU admission with a length of stay greater than or equal to 24 h; 2. Adult patients aged 18 years or older; 3. Complete delirium assessment and essential indicator data. Exclusion criteria were as follows: 1. Patients with dementia; 2. Patients with acute-phase schizophrenia; 3. Patients with complete blindness; 4. Patients with hepatic coma; 5. Patients with an overdose of psychoactive substances; 6. Patients with alcoholism. These exclusions were made because these comorbidities could significantly affect the occurrence of delirium or influence clinical assessments of delirium. The detailed process of participant selection is shown in Figure 1.

### 2.2 Basic data and outcomes

We extracted baseline demographic characteristics, scoring information, comorbidities, laboratory test mean results taken within 24 h of admission, and therapeutic factors during admission in ICU 7 days. The calculation of body mass index (BMI) was using the formula that is weight (kg) divided by height (m) squared. Scoring included GCS (Glasgow Coma Scale), APSIII (Acute Physiology Score), CCI (Charlson Comorbidity Index), SOFA (Sequential Organ Failure Assessment), and nutritional assessment scores which included GNRI (19), PNI (20), TCBI (21), and CONUT score (22). The formulas for calculating these nutritional scores are as follows: GNRI = [1.489 × 10 × serum albumin (g/dL)] + [41.7 × actual weight (kg)/ideal weight (kg)] (ideal weight (kg) = 22 × height (m) squared). The specific value of actual weight (kg)/ideal weight (kg) will be set as one if the actual weight exceeds the ideal body weight. PNI = 10 × serum albumin (g/dL) + 5 × total lymphocyte count (10^9/L); TCBI = serum TG (mg/dL) × TC (mg/dL) × weight (kg)/1,000; The grading criteria for CONUT score is in Supplementary Table S1.

The occurrence of delirium is the primary outcome measure of this study. Assessment Method for the ICU (CAM-ICU) is a validated and user-friendly delirium screening tool at the bedside of the ICU (23). During their ICU stay, a “positive” CAM-ICU assessment result defines the occurrence of ICU delirium. Additionally, secondary outcome measures include in-hospital mortality, total length of hospital stay, and survival days after discharge hospital. We estimated the patients’ survival days based on their final follow-up time.

### 2.3 Preliminary data processing

For the four nutritional indicators (GNRI, PNI, CONUT score, and TCBI), we conducted four separate ROC (Receiver Operating Characteristic) curve analyses to determine the optimal cut-off points for predicting the occurrence of delirium. Based on the optimal cut-off points for the four nutritional scores, the included cases were categorized into low and high groups for each score. The specific grouping results are as follows: the low group for GNRI is ≤85.63 and the high group is >85.63; for PNI, the low group is ≤42.7 and the high group is >42.7; for CONUT score, the low group is <2.5 and the high group is ≥2.5; for TCBI, the low group is <1100.1 and the high group is ≥1100.1. The area under the ROC curve (AUC) for each nutrient indicator individually predicted delirium is as follows: 0.53 (95% CI: 0.47–0.59) for GNRI, 0.62 (95% CI: 0.56–0.68) for PNI, 0.64 (95% CI: 0.58–0.70) for CONUT score, and 0.54 (95% CI: 0.47–0.60) for TCBI.

### 2.4 Statistical analysis

All data analysis in this study used R (version 4.3.2) language. If the two-tailed *p*-value of the statistical result was less than 0.05 on both sides, it was significant. At first, we filtered out variables that were missing exceeding 30%. The specific details of data missingness can be found in Supplementary Table 2. Subsequently, the random forest imputation (rf imputation) method from the “mice” package was applied to impute the remaining missing variables. Next, we used the Shapiro–Wilk test to examine whether the variables exhibited a normal distribution. Continuous variables were presented as mean ± standard deviation for normally distributed data, and as the Median (Interquartile Range) for non-normally distributed data; categorical variables were presented as numbers and percentages (%). Variables were compared using T-tests for normally distributed continuous variables and non-parametric tests (Mann–Whitney U or Kruskal-Wallis test) for nonparametric variables. A comparison of categorical variables was conducted using Fisher’s exact test.

We draw histograms of frequency distribution to visualize the distribution of nutrient scores among the study participants. Subsequently, we used the Spearman rank correlation coefficient to assess the rough association between different nutrient scores and delirium. In addition, we used the “rms” package in R to perform restricted cubic spline (RCS) analysis, further exploring the specific relationship between nutritional scores and delirium. Single-factor and multivariate stepwise logistic regression models were used to investigate the independent relationship between the four nutrient scores and ICU delirium. The advantage of the multivariate stepwise logistic regression model lies in its ability to identify features that make an outstanding contribution, enhance model accuracy, and streamline interpretation. To avoid multicollinearity, variables with variance inflation factor (VIF) greater than or equal to 10 were excluded. Odds ratios (OR) and 95% confidence intervals (CI) were both used to describe the strength of the association between exposure factors and delirium. We constructed three models: an unadjusted model, a partially adjusted model, and a fully adjusted model. Covariates were selected based on prior literature (24) and univariate variables that were statistically significant. Subsequently, the area under the receiver operating characteristic curve (AUC) was employed to compare and evaluate the performance of the four nutrient-based regression models in predicting delirium. At last, we conducted a subgroup analysis using adjusted multivariable regression models to assess whether the impact of malnutrition on ICU delirium occurrence exhibits heterogeneity within stratified covariates, as well as examining the interaction between nutritional score and stratified covariates.

## 3 Results

### 3.1 Baseline characteristics

In our study, 319 participants were included, with 54.9% (175 individuals) of patients experiencing delirium during their ICU stay (Table 1). The delirium group had poorer nutritional status compared to the non-delirium group, with lower PNI scores (*p* < 0.05) and higher CONUT scores (*p* < 0.05). The SOFA score (*p <* 0.05) and APS-III score (*p* < 0.05) showed higher disease severity in patients with delirium. Furthermore, in terms of outcomes, delirious patients had a higher in-hospital mortality rate (*p <* 0.05), more hospital stay days (*p* < 0.05), and a shorter average survival time (*p* < 0.05) compared to the non-delirium group.

**Table 1:** Comparison of general clinical data between delirium and non-delirium patients.

Characteristic	Overall (*n* = 319)	Non-delirium (*n* = 144)	Delirium (*n* = 175)	*P*-value
Demographics				
Age, years	64.00 [54.00, 73.00]	64.00 [54.00, 74.00]	64.00 [53.00, 72.50]	0.97
Gender (%)				0.99
Male	125 (39.2)	57 (39.6)	68 (38.9)	
Female	194 (60.8)	87 (60.4)	107 (61.1)	
Race (%)				<0.05
White	179 (56.1)	94 (65.3)	85 (48.6)	
Black	23 (7.2)	9 (6.2)	14 (8.0)	
Caucasian	8 (2.5)	6 (4.2)	2 (1.1)	
Other	109 (34.2)	35 (24.3)	74 (42.3)	
Weight (kg)	83.40 [68.85, 99.75]	85.50 [68.15, 103.25]	82.40 [69.70, 96.50]	0.27
Height (cm)	170.00 [163.00, 178.00]	170.00 [163.00, 178.25]	170.00 [163.00, 178.00]	0.70
BMI	28.30 [24.58, 33.59]	28.77 [24.68, 34.09]	27.82 [24.49, 32.59]	0.32
Scores				
GCS	15.00 [15.00, 15.00]	15.00 [15.00, 15.00]	15.00 [14.00, 15.00]	<0.05
APS-III	42.00 [30.00, 61.00]	34.50 [25.00, 49.25]	50.00 [35.50, 70.00]	<0.05
CCI	5.00 [3.00, 7.00]	5.00 [3.00, 7.00]	5.00 [3.00, 7.00]	0.13
SOFA	2.00 [0.00, 4.00]	1.00 [0.00, 3.25]	2.00 [0.00, 5.00]	<0.05
GNRI	95.30 [86.37, 101.26]	95.30 [87.61, 101.26]	93.82 [83.53, 100.28]	0.38
PNI	41.91 [35.15, 47.38]	43.75 [37.68, 48.90]	40.65 [33.15, 45.90]	<0.05
CONUT score	4.00 [2.00, 7.00]	3.00 [1.00, 5.25]	5.00 [3.00, 8.00]	<0.05
TCBI	1437.15 [848.54, 2440.60]	1470.63 [883.63, 2462.50]	1308.31 [818.80, 2319.65]	0.29
Comorbidities				
MI (%)				<0.05
No	222 (69.6)	91 (63.2)	131 (74.9)	
Yes	97 (30.4)	53 (36.8)	44 (25.1)	
CHF (%)				<0.05
No	225 (70.5)	91 (63.2)	134 (76.6)	
Yes	94 (29.5)	53 (36.8)	41 (23.4)	
CVD (%)				<0.05
No	198 (62.1)	101 (70.1)	97 (55.4)	
Yes	121 (37.9)	43 (29.9)	78 (44.6)	
COPD (%)				0.55
No	267 (83.7)			
Yes	52 (16.3)	21 (14.6)	31 (17.7)	
Diabetes (%)				<0.05
No	228 (71.5)	92 (63.9)	136 (77.7)	
Yes	91 (28.5)	52 (36.1)	39 (22.3)	
Paraplegia (%)				<0.05
No	247 (77.4)	120 (83.3)	127 (72.6)	
Yes	72 (22.6)	24 (16.7)	48 (27.4)	
Renal disease (%)				0.87
No	268 (84.0)	122 (84.7)	146 (83.4)	
Yes	51 (16.0)	22 (15.3)	29 (16.6)	
Liver disease (%)				<0.05
No	281 (88.1)	136 (94.4)	145 (82.9)	
Yes	38 (11.9)	8 (5.6)	30 (17.1)	
Laboratory tests				
White blood cell (10^9^/L)	10.50 [7.70, 14.75]	9.70 [7.40, 13.03]	11.00 [7.90, 16.05]	<0.05
Red blood cell (10^9^/L)	4.04 [3.37, 4.64]	4.20 [3.48, 4.67]	3.97 [3.29, 4.64]	0.07
Hemoglobin (g/dL)	12.20 [10.10, 13.80]	12.40 [10.47, 13.83]	12.00 [9.65, 13.70]	0.15
Mean corpuscular hemoglobin (pg)	30.20 [28.80, 31.80]	30.00 [28.58, 31.42]	30.30 [28.95, 32.10]	0.08
Platelet (10^9^/L)	192.00 [143.00, 261.50]	201.50 [165.50, 266.75]	182.00 [110.00, 253.00]	<0.05
INR	1.20 [1.10, 1.50]	1.20 [1.10, 1.40]	1.30 [1.10, 1.60]	<0.05
PT (s)	13.30 [11.90, 15.95]	12.75 [11.70, 15.12]	13.70 [12.05, 16.95]	<0.05
Glucose (mg/dL)	125.00 [106.00, 164.00]	125.00 [106.00, 163.25]	124.00 [106.00, 164.50]	0.89
Sodium (mEq/L)	138.00 [135.00, 141.00]	138.50 [136.75, 141.00]	138.00 [134.00, 141.50]	0.49
Potassium (mEq/L)	4.10 [3.70, 4.60]	4.10 [3.70, 4.50]	4.10 [3.70, 4.70]	0.75
Calcium (mg/dL)	8.60 [7.90, 9.10]	8.75 [8.28, 9.22]	8.50 [7.80, 9.00]	<0.05
Chloride (mEq/L)	101.00 [98.00, 105.00]	102.00 [99.00, 105.00]	101.00 [97.00, 105.50]	0.30
Creatinine (mg/dL)	1.00 [0.80, 1.70]	1.00 [0.70, 1.40]	1.10 [0.80, 1.80]	0.05
Albumin (g/dL)	3.60 [3.00, 4.00]	3.70 [3.20, 4.03]	3.50 [2.80, 3.90]	<0.05
Blood urea nitrogen (mg/dL)	20.00 [13.50, 30.50]	18.00 [13.00, 27.00]	20.00 [14.00, 33.50]	0.20
Total cholesterol (mg/dL)	143.00 [105.00, 178.00]	147.00 [118.75, 187.00]	138.00 [95.50, 168.00]	<0.05
Triglycerides (mg/dL)	122.00 [85.50, 190.50]	120.50 [81.00, 179.25]	126.00 [88.00, 196.50]	0.35
PH	7.39 [7.33, 7.44]	7.40 [7.35, 7.44]	7.38 [7.29, 7.44]	<0.05
PO_2_	78.00 [52.00, 162.00]	74.00 [46.25, 165.00]	84.00 [57.50, 134.50]	0.46
PCO_2_	39.00 [35.00, 45.00]	40.00 [35.00, 46.00]	39.00 [34.00, 45.00]	0.47
Eosinophils (10^9^/L)	0.03 [0.00, 0.12]	0.06 [0.01, 0.14]	0.01 [0.00, 0.09]	<0.05
Lymphocyte count (10^9^/L)	1.09 [0.69, 1.71]	1.31 [0.83, 1.78]	0.93 [0.62, 1.62]	<0.05
Neutrophils (10^9^/L)	7.94 [5.38, 11.98]	7.30 [5.16, 10.42]	8.55 [6.12, 13.09]	<0.05
Therapeutic factor				
Ventilation status (%)				<0.05
No Oxygen	52 (16.3)	40 (27.8)	12 (6.9)	
Non-invasive	148 (46.4)	36 (25.0)	112 (64.0)	
Invasive	119 (37.3)	68 (47.2)	51 (29.1)	
Used propofol (%)				<0.05
Unused	127 (39.8)	97 (67.4)	30 (17.1)	
Used	192 (60.2)	47 (32.6)	145 (82.9)	
Used vasoactive agents (%)				<0.05
Unused	163 (51.1)	92 (63.9)	71 (40.6)	
Used	156 (48.9)	52 (36.1)	104 (59.4)	
Outcomes				
Hospital deaths (%)				<0.05
No	269 (84.3)	131 (91.0)	138 (78.9)	
Yes	50 (15.7)	13 (9.0)	37 (21.1)	
Hospital days	4.93 [2.56, 9.94]	2.71 [1.73, 4.60]	8.78 [4.78, 15.39]	<0.05
Survival days	375.00 [368.00, 387.00]	372.50 [368.00, 379.00]	380.00 [369.00, 391.50]	<0.05

Data are presented as N (%) or Median (IQR). BMI, body mass index; CCI, Charlson comorbidity index; GCS, Glasgow coma score; APS-III, acute physiology score III; SOFA, sequential organ failure assessment; GNRI, geriatric nutritional risk index; PNI, Prognostic Nutritional Index; CONUT score, Controlling Nutritional Status score; TCBI, Triglycerides × Total Cholesterol × Body Weight Index; MI, Myocardial infarct; CHF, Congestive heart failure; CVD, Cerebrovascular disease; COPD, Chronic pulmonary disease; INR, International normalized ratio; PT, Prothrombin time; Non-invasive, Non-invasive Oxygen supplementation; Invasive, Invasive Ventilation.

### 3.2 Association between four nutritional scores and ICU delirium

**Figure 2:**
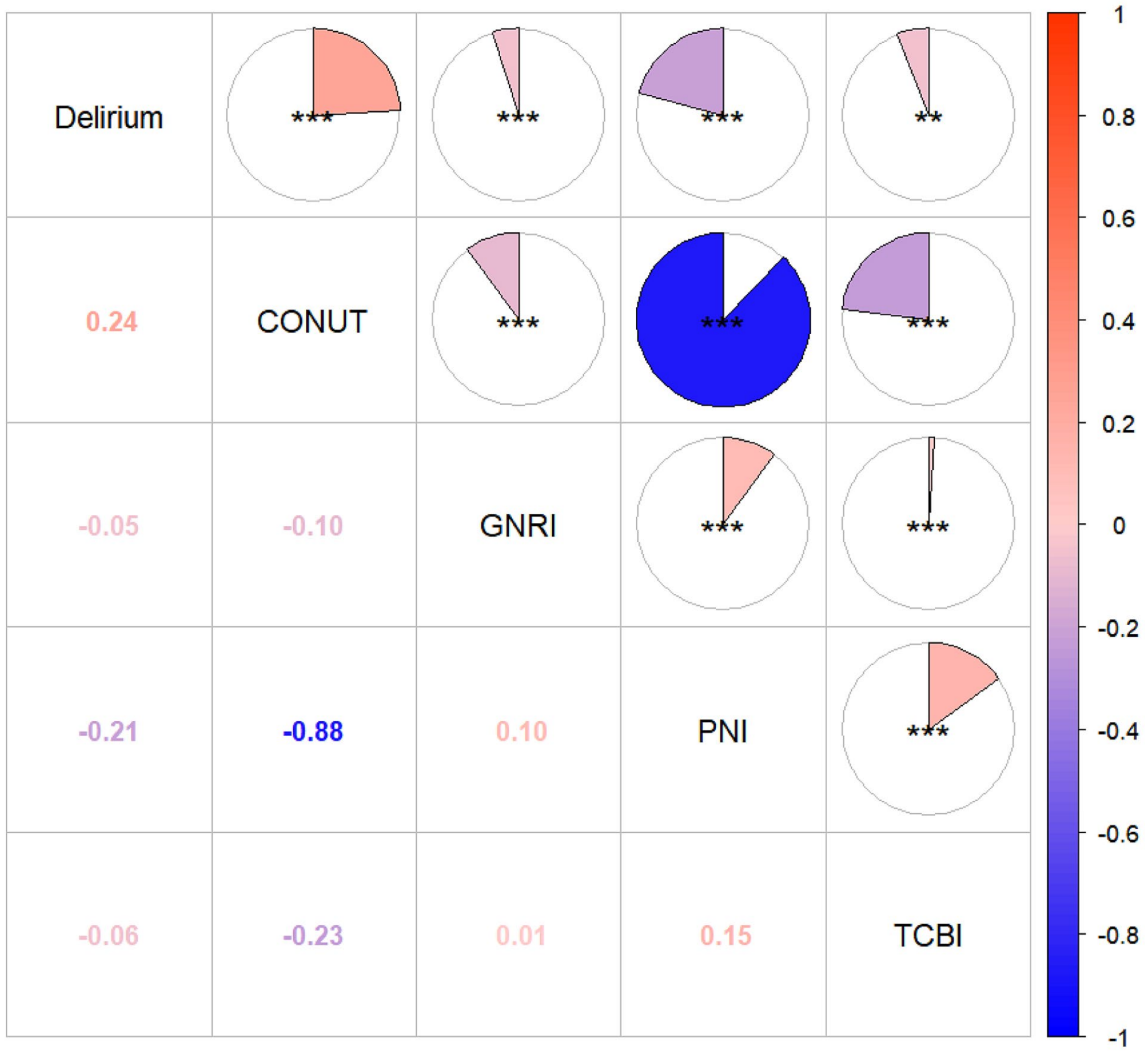
Heat map of correlation between four nutritional scores and delirium. GNRI, geriatric nutritional risk index; PNI, Prognostic Nutritional Index; CONUT score, Controlling Nutritional Status score; TCBI, Triglycerides × Total Cholesterol × Body Weight Index; *p ≤ 0.05; **p ≤ 0.01; ***p ≤ 0.001.

**Figure 3:**
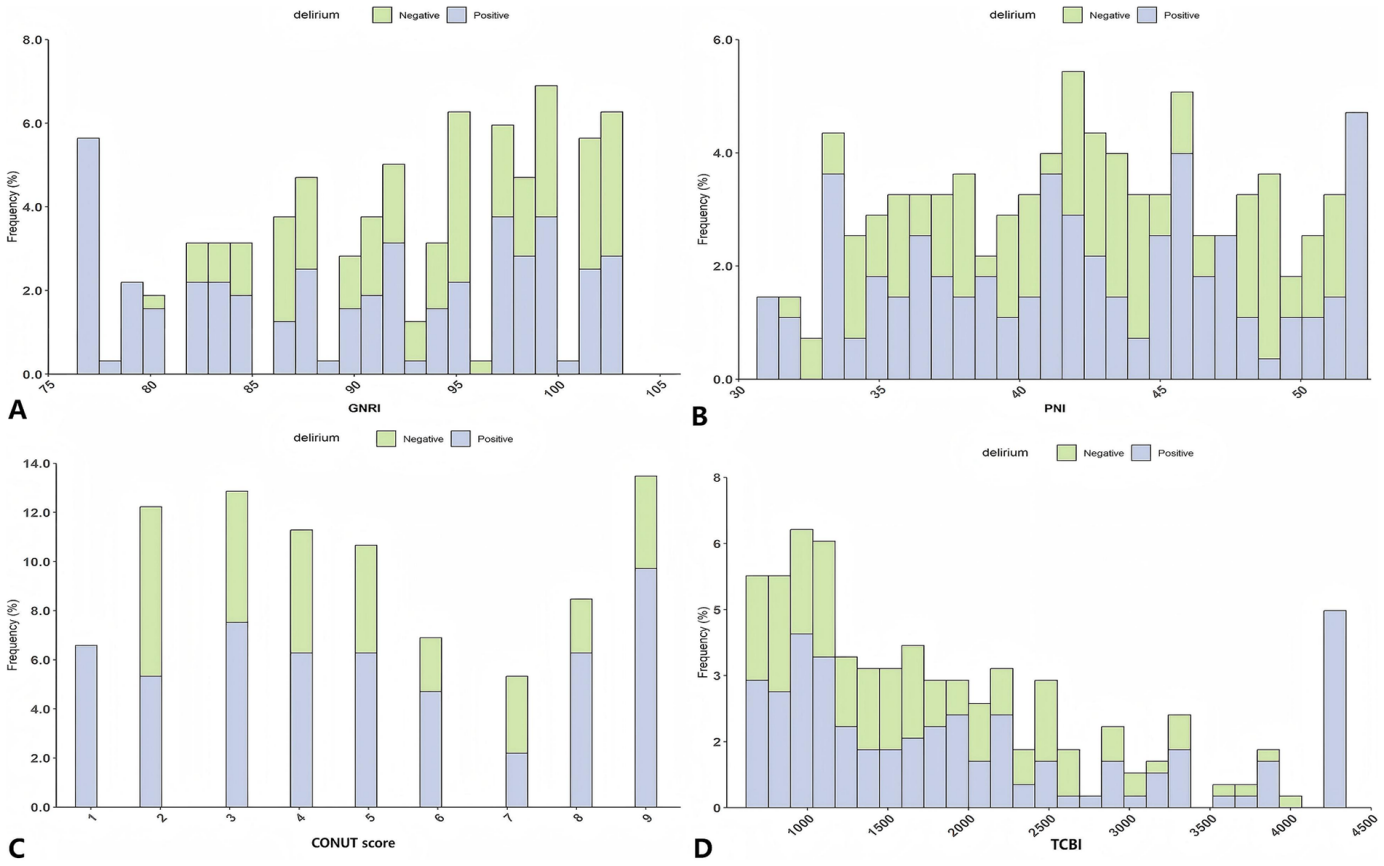
Analysis of the relationship between nutrition and delirium: frequency distribution histogram. **(A)** GNRI; **(B)** PNI; **(C)** CONUT score; **(D)** TCBI; GNRI, geriatric nutritional risk index; PNI, Prognostic Nutritional Index; CONUT score, Controlling Nutritional Status score; TCBI, Triglycerides × Total Cholesterol × Body Weight Index.

**Figure 4:**
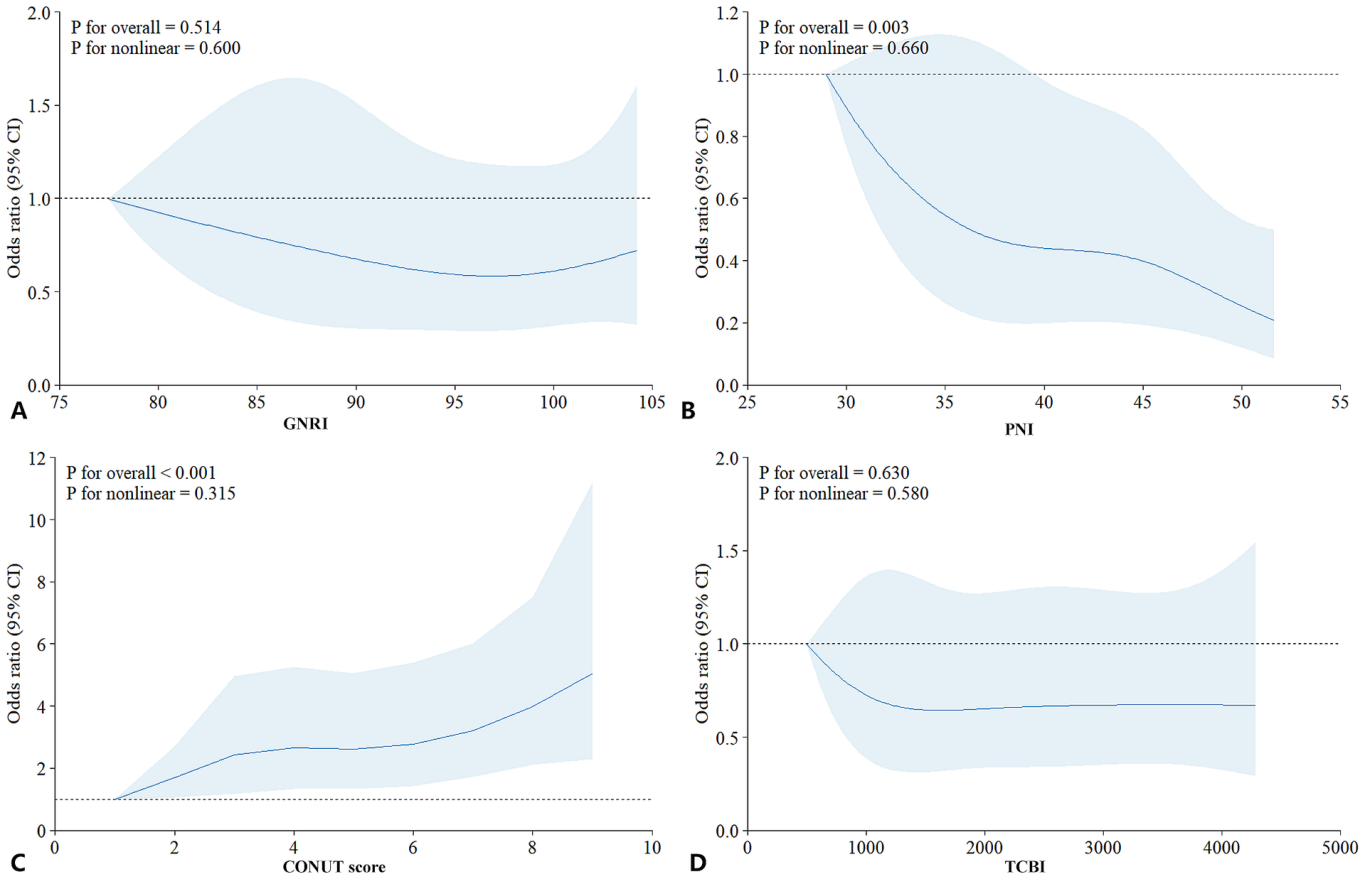
Analysis of the relationship between nutrition and delirium: restricted cubic spline plot. **(A)** GNRI; **(B)** PNI; **(C)** CONUT score; **(D)** TCBI; GNRI, geriatric nutritional risk index; PNI, Prognostic Nutritional Index; CONUT score, Controlling Nutritional Status score; TCBI, Triglycerides × Total Cholesterol × Body Weight Index.

Correlation analyses (Figure 2 correlation heat map) showed that GNRI (*r* = −0.05, *p =* 0.05), PNI (*r* = −0.21, *p* < 0.05), and TCBI (*r* = −0.06, *p* < 0.05) were all negatively correlated with delirium occurrence. The CONUT score showed a positive correlation with the occurrence of delirium (*r* = 0.24, *p* < 0.05). In PNI, GNRI, and TCBI, as the scores increased, the occurrence of delirium showed a decreasing trend, while in the CONUT score, the trend was the opposite (Figures 3A–D). The restricted spline curves showed a potential linear relationship between PNI and CONUT score with delirium (Figures 4B,C). For PNI, as the predicted PNI increased, the risk of delirium decreased, but the risk was relatively stable in the PNI range of 35–45 (overall *p* < 0.05, nonlinear *p* = 0.66). For the CONUT score, as the predicted CONUT score increased, the risk of delirium showed a steadily rising trend (overall *p* < 0.05, nonlinear *p* = 0.32).

Subsequently, single-factor (Supplementary Table S3) and multivariate stepwise logistic regression (Table 2) analyses were conducted In single-factor regression, patients with lower GNRI (≤85.6), lower PNI (≤42.7), and higher CONUT score (≥2.5) had an increased risk of ICU delirium (*p* < 0.05). TCBI did not show a significant correlation with delirium (*p* = 0.09) and was therefore excluded from the multivariate regression model. In addition, race, GCS, APSIII, SOFA, platelets, calcium, creatinine, eosinophils, arterial blood PH, ventilation status, use of propofol, and vasoactive agents were also significantly associated with delirium.

**Table 2:** Multifactor stepwise regression.

Nutritional scores	GNRI		PNI		CONUT score	
	≤85.626	>85.626	≤42.650	>42.650	<2.5	≥2.5
Model 1						
OR (95%CI)	Reference	1.70 (1.01–2.91)	Reference	2.42 (1.54–3.82)	Reference	2.65 (1.63–4.35)
*P*-value		<0.05		<0.05		<0.05
AUC (95%CI)	0.55 (0.50–0.60)		0.61 (0.55–0.66)		0.60 (0.55–0.65)	
*P*-value	<0.05		<0.05		<0.05	
Model 2						
OR (95%CI)	Reference	1.74 (1.02–3.01)	Reference	2.21 (1.51–3.82)	Reference	2.71 (1.65–4.52)
*P*-value		<0.05		<0.05		<0.05
AUC (95%CI)	0.63 (0.57–0.69)		0.66 (0.60–0.72)		0.66 (0.61–0.72)	
*P*-value	<0.05		<0.05		<0.05	
Model 3						
OR (95%CI)	Excluded		Reference	2.04 (1.05–4.03)	Excluded	
*P*-value				<0.05		
AUC (95%CI)			0.87 (0.83–0.91)			
*P*-value			<0.05			

GNRI, geriatric nutritional risk index; PNI, Prognostic Nutritional Index; CONUT score, Controlling Nutritional Status score; TCBI, Triglycerides × Total Cholesterol × Body Weight Index; Data are presented as odds ratio, 95%CI (confidence intervals), and *p*-value; AUC: area under the curve; AIC: Akaike information criterion. Model 1 adjusted for none. Model 2 adjusted for race. Model 3 adjusted for race, GCS, APS-III, SOFA, platelet, calcium, creatinine, PH, eosinophils, ventilation status, propofol, and vasoactive agent substances.

In the adjusted multivariate stepwise logistic regression model 3, only PNI remained significantly associated with delirium occurrence. PNI was an independent predictor of delirium (OR = 2.04, 95% CI: 1.05–4.03, *p* < 0.05) (Table 2). The model’s ROC was 0.87 (95% confidence interval: 0.83–0.91, *p* < 0.05), indicating good accuracy of the model (Supplementary Figure S1).

### 3.3 Subgroup analysis

**Figure 5:**
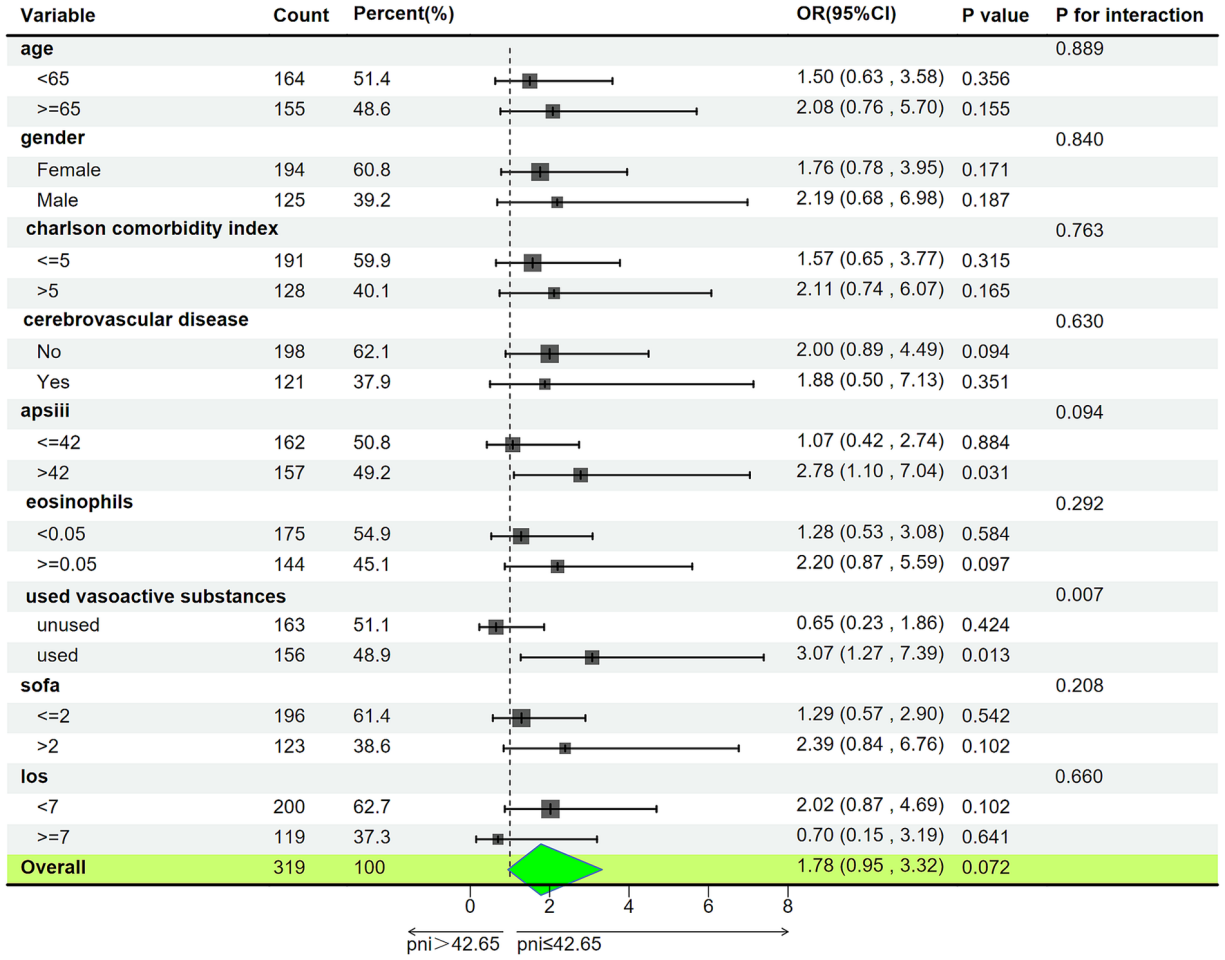
Subgroup analysis of associations between PNI and delirium. APSIII, acute physiology score III; SOFA, sequential organ failure assessment; LOS, Length of Stay (day); PNI, Prognostic Nutritional Index; OR, Odds Ratio; 95% CI, 95% Confidence Interval.

We stratified patients based on age, gender, Charlson Comorbidity Index, history of cerebrovascular disease, APS-III, eosinophils, use of vasoactive drugs, SOFA, and total days of hospitalization (Figure 5). In patients using vasoactive agents, the association between lower PNI and increased risk of delirium was more significant (odds ratio OR = 3.07, 95% confidence interval: 1.27–7.39, interaction *p*-value<0.05), while in the other subgroups, the predictive effect of PNI on delirium occurrence was consistent (interaction *p-*value >0.05).

## 4 Discussion

This study is the first to simultaneously explore the relationship between four nutritional scores (GNRI, TCBI, PNI, and CONUT) and ICU delirium. The main conclusions are as follows: (1) Poor nutritional status is associated with an increased incidence of ICU delirium; (2) Among the four nutritional scores, PNI may be the most efficient independent predictor of ICU delirium, while the correlation between TCBI and ICU delirium is the weakest. Subgroup analysis shows that this relationship is more pronounced in patients using vasoactive substances. Compared to predicting independently with PNI, a multivariable regression model consisting of PNI and other delirium-influencing factors showed a higher predictive value for ICU delirium.

Our findings are consistent with those of most previous studies, confirming that poor nutritional status may be one of the risk factors for delirium. In fact, on the one hand, the brain is a metabolically active organ with high nutritional demands, and the lack of nutrients may increase the risk of delirium (6, 25). On the other hand, the development of delirium may further lead to decreased appetite or swallowing difficulties in patients, increasing the risk of worsening nutritional status (26). Therefore, nutritional status and delirium may interact, and focusing on improving the nutritional levels of critically ill patients is crucial to prevent delirium and further deterioration of nutritional status.

Previous studies showed that various nutritional assessment indicators have the potential ability to predict delirium. To identify the optimal nutritional indicators for predicting delirium, we concurrently selected and compared four nutritional assessment tools: GNRI, TCBI, PNI, and COUNT score. These scores are from objective and easily measurable health parameters. Compared to questionnaire-based nutritional screening, they avoid assessment biases caused by inaccurate responses, making them more suitable for the tense environment of the ICU and patients with altered mental status. The results show that PNI and the CONUT score are more strongly correlated with delirium, significantly higher than GNRI and TCBI. There is an L-shaped relationship between PNI and delirium, while the correlation presented in the CONUT score is inverse. However, after adjusting other covariates, we found that only PNI could independently predict the occurrence of delirium in the ICU. Fan et al. (27) found that the PNI score and all-cause mortality in the general population also demonstrated an L-shaped relationship, as well as it was the most predictive nutritional index. Delirium serves as an early warning sign for adverse outcomes in patients (28), but whether PNI can also directly predict the prognosis of critically ill patients requires further clarification, especially for those who have experienced delirium. As an immune nutritional indicator, PNI was initially used to assess the preoperative nutritional status of patients with gastrointestinal tumors and to achieve risk prediction for postoperative complications (20). The PNI has been widely used for prognostic assessment in various cancer patients (29) and is now also used to predict the occurrence or prognosis of postoperative, cardiovascular disease, and emergency delirium in patients (15, 30–33). PNI evaluates both serum albumin and lymphocytes, and these two biochemical parameters may have significant connections to the mechanism of delirium. Although the physiological mechanism of delirium is not yet understood, the neuroinflammatory hypothesis suggests that there is a link between the brain and the peripheral immune system in the human body, and peripheral inflammation can directly affect brain function, potentially leading to the occurrence of delirium (34). Serum albumin is an essential indicator of malnutrition in patients and plays a vital role in immune regulation, possessing anti-inflammatory and antioxidant properties (35, 36). Lymphocytes play a central role in the body’s immune response. Recent studies have indicated that CD4 T cell subsets may be potential biological markers for delirium (37). In addition, the neutrophil-to-lymphocyte ratio (NLR) is an emerging marker representing systemic inflammation, which may have better sensitivity for early delirium than lymphocytes alone (38–40). More research is needed to explore the specific physiological mechanisms linking albumin and lymphocytes with delirium. Interestingly, in patients using vasoactive substances, the risk of delirium with low PNI is significantly higher than in non-users. This may be related to vasopressor-induced deterioration of nutritional status. Vasopressors can cause redistribution of blood flow and vasoconstriction, potentially reducing blood flow to the gastrointestinal tract and liver (41, 42). This could lead to decreased albumin synthesis and impaired nutrient absorption. On the one hand, existing studies have shown that vasopressors (such as norepinephrine) may disrupt immune balance, enhancing the release of pro-inflammatory cytokines, which in turn can trigger immune dysregulation or immune suppression (43). Therefore, it is recommended to carefully assess the nutritional status of patients receiving vasopressor therapy, particularly at high doses (41).

The CONUT score (21) and PNI both include the evaluation of serum albumin and lymphocytes. The predictive value of PNI is superior to that of the CONUT score, possibly because PNI uses the original continuously measured values, reducing the loss of information. On the other hand, the CONUT score also evaluates the levels of total cholesterol, which we speculate may be an interfering factor affecting the predictive accuracy. Based on limited research reports on the correlation between cholesterol and delirium, although total cholesterol is not an independent predictor of delirium (31), high-density lipoprotein cholesterol (HDL-C) (44) and low-density lipoprotein cholesterol (LDL-C) (45) may be protective and risk factors for delirium, respectively. Compared to total cholesterol levels, HDL-C and LDL-C appear to have closer correlations with delirium. However, the delirium populations included in these studies are heterogeneous, and the specific relationship between cholesterol and delirium deserves further investigation. TCBI is a novel nutritional index calculated based on serum triglycerides, total cholesterol, and body weight. Previous studies have demonstrated that TCBI can effectively predict the prognosis of critically ill patients, coronary heart disease, acute decompensated heart failure, and the general population (21). In this study, however, TCBI is not an ideal nutritional assessment tool for predicting delirium, possibly because it does not accurately reflect the body’s immune response and inflammation levels like PNI. Significantly, Huang and others (46) found and validated the independent positive correlation between the triglyceride-glucose index (TyG) and severe delirium in a multicenter sample. This suggests that the onset of delirium may be related to insulin resistance-induced dyslipidemia, whereas lipid metabolism alone may not sufficiently reflect the nutritional status of delirium patients. Wei et al.’s study (7) found a non-linear relationship between GNRI and delirium in critically ill elderly individuals, it could predict delirium occurrence in elderly ICU patients well. Unfortunately, our study did not find this relationship, which may be related to the fact that our study subjects were not solely elderly. Additionally, obtaining accurate weight and height of critically ill patients in clinical practice can be challenging. In conclusion, identifying appropriate nutritional indicators to recognize and address malnutrition in critically ill patients may be one of the crucial strategies for the prevention and management of delirium in the ICU.

There are several limitations in this study. (1) This is a single-center retrospective study, which prevents causal inference regarding the relationship between nutritional status and delirium. Additionally, we only collected and recorded the average nutritional levels of patients within 24 h of admission, while dynamic data monitoring could more accurately reflect changes in nutritional status and potentially provide better predictive value for delirium. Therefore, future studies should establish larger-scale, prospective longitudinal studies to further validate and explore these findings. (2) The study included a diverse group of ICU patients, which, while enhancing the generalizability of PNI across various ICU settings, may introduce bias due to the differences in the patients’ primary diseases. As different diseases may have distinct effects on patients’ nutritional status, future studies should explore supplementary nutritional indices to provide a more comprehensive and accurate assessment of nutritional status in patients across different ICU environments. (3) Although many important confounders were adjusted for, there remains a possibility that some variables affecting the results were not included due to the limitations of the database. For example, other analgesic and sedative drugs, as well as antidepressants, could potentially influence the occurrence of delirium.

In conclusion, our study demonstrated a correlation between delirium in ICU patients and four objective nutritional scores (GNRI, PNI, TCBI, and CONUT). Among these, PNI exhibited the highest independent predictive value for delirium occurrence in critically ill patients. PNI has the advantage of simplicity and easy availability, making it a suitable nutritional assessment tool for evaluating delirium risk upon initial ICU admission. Clinicians can use PNI to assess patients’ nutritional and immune levels and implement targeted interventions to reduce the risk of delirium. However, these findings require validation in larger cohorts to enhance their generalizability.

## Data Availability

Publicly available datasets were analyzed in this study. This data can be found at: https://physionet.org/content/mimiciv/2.2/%3c/b.
