# Supplementary material for: Comparative analysis of four nutritional scores in predicting delirium in ICU patients
Source: Front Nutr. 2025 Jul 22;12:1482150. doi: 10.3389/fnut.2025.1482150 (PMC12321548; doi:10.3389/fnut.2025.1482150)
Supplement: Supplementary file 1 [file Table_1.docx]

# Supplementary Table

**Supplementary Table 1** Parameters for assessment of the CONUT Score.

| **Table S1. Evaluation of CONUT score** | | | | |
| --- | --- | --- | --- | --- |
| Parameter | Score | | | |
| Serum albumin (g/dL) | ≥3.5 | 3.0-3.4 | 2.5-2.9 | <2.5 |
| Albumin score | 0 | 2 | 4 | 6 |
| Total cholesterol (mg/dL) | ≥180 | 140-179 | 100-139 | <100 |
| Cholesterol score | 0 | 1 | 2 | 3 |
| Total lymphocytes (10^9/L) | ≥1.600 | 1.200-1.599 | 0.800-1.199 | <0.800 |
| Lymphocyte score | 0 | 1 | 2 | 3 |

**Supplementary Table 2** Summary of Missing Data

| **Characteristic** | **N (%)** |
| --- | --- |
| SOFA | 1 (0.3%) |
| Red blood cell | 2 (0.6%) |
| Hemoglobin | 2 (0.6%) |
| Mean corpuscular hemoglobin | 2 (0.6%) |
| INR | 7 (2.2%) |
| PT | 7 (2.2%) |
| Glucose | 14 (4.4%) |
| Sodium | 10 (3.1%) |
| Potassium | 11 (3.5%) |
| Calcium | 76 (23.8%) |
| Chloride | 10 (3.1%) |
| Creatinine | 10 (3.1%) |
| Blood urea nitrogen | 3 (0.9%) |
| PH | 62 (19.4%) |
| PO_2_ | 62 (19.4%) |
| PCO_2_ | 62 (19.4%) |

SOFA, sequential organ failure assessment; INR, International normalized ratio; PT, Prothrombin time;

**Supplementary Table 3** Univariate regression

| **valuables** | **OR (95%CI)** | ***P-* Valve** |
| --- | --- | --- |
| Race |  |  |
| White | Reference |  |
| Black | 1.72 (0.71-4.18) | 0.23 |
| Caucasian | 0.37 (0.07-1.88) | 0.23 |
| Other | 2.34 (1.42-3.85) | <0.05 |
| GCS | 0.50 (0.34-0.74) | <0.05 |
| APS-III | 1.04 (1.02-1.05) | <0.05 |
| SOFA | 1.11 (1.02-1.20) | <0.05 |
| Platelet | 1.00 (0.99-1.00) | <0.05 |
| Calcium | 0.65 (0.48-0.88) | <0.05 |
| Creatinine | 1.44 (1.05-1.98) | <0.05 |
| PH | 0.02 (0.00-0.33) | <0.05 |
| Eosinophils | 0.02 (0.00-0.32) | <0.05 |
| Ventilation status |  |  |
| No Oxygen | Reference |  |
| Non-invasive Oxygen supplementation | 10.37 (4.92-21.88) | <0.05 |
| Invasive Ventilation | 2.50 (1.19-5.24) | <0.05 |
| Used propofol |  |  |
| Unused | Reference |  |
| Use | 9.98 (5.90-16.89) | <0.05 |
| Used vasoactive agents |  |  |
| Unused | Reference |  |
| Use | 2.59 (1.64-4.08) | <0.05 |
| GNRI |  |  |
| ≤85.626 | Reference |  |
| ＞85.626 | 1.70 (1.01-2.88) | 0.05 |
| PNI |  |  |
| ≤42.650 | Reference |  |
| ＞42.650 | 2.42(1.54-3.0) | <0.05 |
| TCBI |  |  |
| <1100.145 | Reference |  |
| ≥1100.145 | 1.49 (0.94-2.35) | 0.09 |
| CONUT score |  |  |
| <2.5 | Reference |  |
| ≥2.5 | 2.65(1.63-4.32) | <0.05 |

GCS, Glasgow coma score; APS-III, acute physiology score III; SOFA, sequential organ failure assessment; GNRI, geriatric nutritional risk index; PNI, Prognostic Nutritional Index; CONUT score, Controlling Nutritional Status score; TCBI, Triglycerides × Total Cholesterol × Body Weight Index; 95%CI: 95% confidence interval.

# Supplementary Figures

**Figure S1 The Prognostic Nutritional Index multifactor regression model was used to predict the receiver operating characteristic curve (ROC) of delirium.**


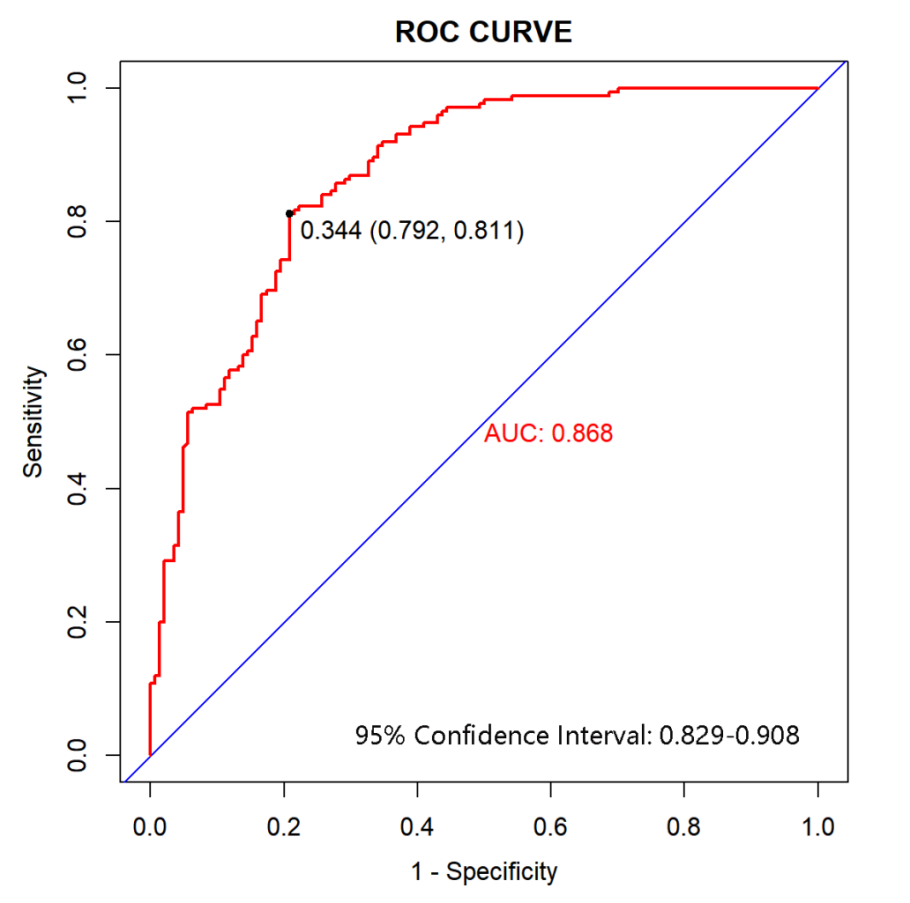


AUC: area under the curve. The model adjusted for race, GCS, APS-III, SOFA, chronic disease, platelet, calcium, creatinine, PH, eosinophils, ventilation status, used propofol, and used vasoactive agent substances.
